# IGFBP-2 and -3 co-ordinately regulate IGF1 induced matrix mineralisation of differentiating human dental pulp cells

**DOI:** 10.1016/j.scr.2016.09.026

**Published:** 2016-11

**Authors:** Hanaa Alkharobi, Aishah Alhodhodi, Yousef Hawsawi, Hasanain Alkafaji, Deirdre Devine, Reem El-Gendy, James Beattie

**Affiliations:** a Division of Oral Biology, Leeds School of Dentistry, Level 7 Wellcome Trust Brenner Building, St James University Hospital, University of Leeds, Leeds LS9 7TF, United Kingdom; b Dept. of Medical Breast Oncology, MD Anderson Cancer Research Centre, University of Texas, Houston, United States; c Dept. of Oral Pathology, Faculty of Dentistry, Suez Canal University, Ismailia, Egypt

**Keywords:** Dental pulp cells, Differentiation, IGF axis

## Abstract

Human dental pulp cells (DPCs), which are known to contain a subset of stem cells capable of reforming a dentin and pulp-like complex upon in vivo transplantation, were isolated from third molars of three healthy donors and differentiated to a matrix mineralisation phenotype using by culture in dexamethasone and l-ascorbic acid. qRT-PCR analysis of insulin-like growth factor ( IGF) axis gene expression indicated that all genes, except insulin-like growth factor 1 (IGF1) and insulin-like growth factor binding protein-1 ( IGFBP-1), were expressed in DPCs. During differentiation upregulation of insulin-like growth factor binding protein-2 (IGFBP-2) and downregulation of insulin-like growth factor binding protein-3 (IGFBP-3) expression was observed. Changes in IGFBP-2 and IGFBP-3 mRNA expression were confirmed at the protein level by ELISA of DPC conditioned medium functional analysis indicated that IGF1 stimulated the differentiation of DPCs and that the activity of the growth factor was enhanced by pre-complexation with IGFBP-2 but inhibited by pre-complexation with IGFBP-3. Therefore changes in IGFBP-2 and -3 expression during differentiation form part of a co-ordinated functional response to enhance the pro-differentiative action of IGF1 and represent a novel mechanism for the regulation of DPC differentiation.

## Highlights

• IGF axis expression is characterised in human dental pulp cells (DPCs).
• Reciprocal changes are identified in expression of IGFBP-2 and -3 during matrix mineralisation of DPCs
• This forms part of a functional co-ordinated response during the IGF1 stimulated differentiation of DPCs.

## 1 Introduction

The increasing demand for alternatives to bone grafting as a method of treating critical bone defects has led to a growing interest in cell based regenerative therapies and selection of appropriate cell sources for bone tissue engineering is a key research area. Recently, cells derived from human dental pulp (DPCs) have been characterised as an accessible source of multipotent stem cells which can be differentiated to a matrix mineralised phenotype. In vivo transplant studies with DPCs indicate a tissue formation whose phenotype most resembles dentin in structure although the processes of dentinogenesis and osteogenesis share regulatory mechanisms and gene expression profiles (Gronthos et al., 2002). Subsequently stem cells have been isolated from other tissue niches within the oral cavity including periodontal ligament (PDLSCs) (Seo et al., 2004), apical papilla (SCAP) (Sonoyama et al., 2006), dental follicle (DFSCs) (Morsczeck et al., 2005), and exfoliated deciduous teeth (SHED) (Gronthos et al., 2000a). As dental tissues develop from oral ectoderm and neural crest derived mesenchyme, they contain pluripotent stem cell populations which display a developmental potential similar to embryonic stem cells (ESCs) and are able to differentiate into several different lineages (Le Douarin and Dupin, 2003, Le Douarin et al., 2004). Typically undifferentiated cells display a fibroblast-like morphology with associated high efficiency for adherent colony formation and high proliferative potential (Kerkis & Caplan, 2011). All of these factors suggest that adult dental tissues may provide a source of material (often discarded in the clinic) to provide multipotent cells for subsequent tissue engineering studies.

Also critical to the success of hard tissue engineering strategies is the selection and use of an appropriate growth factor regimen. Although several growth factors are reported to play an important role in the process of matrix mineralisation, IGF-1 is the most abundant growth factor in bone matrix (Canalis et al., 1993) and several studies have shown that the IGF axis plays an important role in the development and maintenance of skeletal tissues (Delany et al., 2001, Gan et al., 2011, Wong et al., 1990, Yang et al., 2011, Zhang et al., 2002). IGF1 also regulates the osteogenic differentiation of mesenchymal stem cells (MSCs) via mTOR signalling (Xian et al., 2012), BMP9 (Chen et al., n.d.) and MEK-ERK mediated upregulation of the transcriptional co-activator with PDZ-binding motif (TAZ) protein (Xue et al., 2013). These alterations in cell physiology ultimately lead to expression of characteristic osteoblast marker genes, the phenotypic differentiation of osteoblasts and the process of bone accretion (Koch et al., 2005). While some of the molecular details associated with IGF action on osteogenic differentiation are becoming apparent much less information is available regarding the potential functions of the six high affinity IGF binding proteins (IGFBP 1–6) in this process. Such studies are largely limited to the role of IGFBP-4 and IGFBP-5 in mature osteoblasts or bone tissue (Andress, 1995, Andress and Birnbaum, 1992, Durham et al., 1995, Durham et al., 1994) with little information on the role of IGFBPs on the process of differentiation per se.

To further investigate the role of IGFBPs in differentiation we have used DPCs to examine the expression of the IGF axis during the process of matrix mineralisation in these cells. We show that, with the exception of IGF-1 and IGFBP-1, all components of the IGF axis are expressed at moderate to high levels in both basal and mineralising conditions in these cells. We describe consistent and reproducible changes in expression of IGFBP-2 and -3 in cell cultures derived from all donors during differentiation to a mineralising phenotype at both mRNA and protein levels. In addition we demonstrate that IGFBP-2 and IGFBP-3 perform co-ordinated roles as an enhancer or inhibitor respectively of the pro- differentiating activity of IGF1. As IGFBP-2 and -3 have previously been unreported as regulators of matrix mineralisation we believe our data provide a basis for novel further investigations on the role of these two genes in this process and may impact on the design of future hard tissue engineering strategies.

## 2 Materials & methods

### 2.1 Materials

Tissue culture medium α-MEM, FBS and phosphate buffered saline (PBS) were from Lonza (Slough, UK); penicillin/streptomycin (PS), l-glutamine, l-ascorbic acid and dexamethasone, were from Sigma (Dorset, UK). Collagenase (Type I), was from Life Technologies (UK). Tissue culture plastic was from Corning (Amsterdam, Netherlands). Recombinant hIGF1, hIGFBP-2 and hIGFBP-3 were supplied by R&D Systems (Abingdon, UK). All other reagents were of analytical grade or better.

### 2.2 Methods

#### 2.2.1 Tissue culture

Freshly extracted healthy third molars were collected from patients (20–35 years of age) at the dental clinic of Leeds Dental Institute after obtaining informed written consent. Ethical approval and teeth were supplied through the Leeds Dental Institute Tissue Bank (LDI Research Tissue Bank; Ref No. 130111/AH/75). DPCs were isolated from third molars using collagenase enzyme digestion as described previously (Gronthos et al., 2000b, El-Gendy et al., 2012). DPCs at passage 4 were cultured in basal media (α-MEM, supplemented with 20% FBS, 1% penicillin streptomycin (P/S)), and 1% l-glutamine or cultured under mineralisation inducing conditions – (basal media + 10 nM dexamethasone and 50 μg/ml of l-ascorbic acid). Cultures were routinely terminated at 1 and 3 wk. time points for qRT-RCR analysis of gene expression, ELISA and histological staining (ALP and Alizarin Red).

#### 2.2.2 qRT-PCR

RNeasy® Mini Kit (Qiagen, UK) was used to extract and purify mRNA exactly according to the manufacturer's protocol. mRNA quality was confirmed by monitoring A260/280 ratios and 1 μg mRNA was used for first strand cDNA synthesis using High Capacity RNA to cDNA kit (Applied Biosystems, UK) was used according to the manufacturer's protocol. qRT-PCR reactions were conducted in a total volume of 20 μl using TaqMan probes and primers. Expression of each gene was analysed in triplicate in 96 well qRT-PCR plates. Each plate contained a non-template and a reverse transcriptase negative control. qRT-PCR reactions were amplified using the Roche 480 Light Cycler®. Gene expression analysis was carried out using the ∆ Ct method. Ct values of all genes of interest were normalised to the house keeping gene GAPDH. Gene expression under matrix mineralisation conditions was compared to controls cultured under basal conditions using the ΔΔCt method which is plotted as ordinate and indicates fold differences in expression mineralising v basal (El-Gendy et al., 2012). Assay identifiers for TaqMan qRT-PCR reactions are presented in Supplementary Table II.

#### 2.2.3 Elisa

IGFBP-2 and IGFBP-3 concentrations in conditioned media were determined by enzyme-linked immunosorbent assay (ELISA) (R & D Systems, UK) exactly according to the manufacturer's protocol. 1 ml of conditioned medium was collected from basal and mineralising cultures at both 1 (0–7 day conditioning period) and 3 wk (14–21 day conditioning period) time points, centrifuged briefly to remove cell debris and stored at − 80 °C prior to assay. Standard curves for ELISA were linear between 0.0625 and 4 ng/ml (IGFBP-2) and 0.781–50 ng/ml (IGFBP-3). Samples of conditioned medium were appropriately diluted to fall in this region of the standard curve. Data is presented for each individual donor and combined data is presented as a global analysis in Supplementary Figs. 2 and 3.

#### 2.2.4 In vitro bioassay

ALP activity was assayed as described previously (Kim et al., 2013, Kim et al., 2011) with some modifications. Briefly DPCs were grown to confluence and incubated with IGF1 ± IGFBP-2/3 in mineralising medium. Medium was changed at days 4, 7, 10 and cultures were terminated at day 14. Cells were lysed by the addition of 200 μl 0.1% Triton X-100 followed by three cycles of freeze-thawing. Lysates were centrifuged (5 min; 10,000*g*) and 20 μl was assayed for ALP activity. Data are presented as nmol *p*-nitrophenol (pNP) formed per μg DNA.

### 2.3 Statistics

Data were analysed for significance using Student's unpaired *t*-test (Graph Pad 6.0). Significance was reported at p < 0.05.

## 3 Results

Three donors were used to isolate dental pulp from healthy third molars. Donor profile is shown in Supplementary Table I. Dental pulp cells were treated with mineralisation medium for 1 and 3 wk. In order to confirm differentiation we examined the expression of alkaline phosphatase (*ALPL*), runt related transcription factor 2 (*Runx2*) and osteocalcin (*OCN*) under both basal and mineralising culture conditions. *ALPL* is an early marker of differentiation and was upregulated approximately 3-fold at 1 wk with expression levels returning to basal levels at 3 wk (p < 0.05 1 wk v 3 wk). *Runx2* is a key transcription factor regulating matrix mineralisation and was upregulated at both time points although there was a tendency toward higher levels of expression at 3 wk (3-fold) compared to 1 wk (1.5-fold). *OCN* is a later marker of differentiation and is upregulated approximately 2-fold at 3 wk compared to 1 wk (p < 0.01 1 wk v 3 wk) Supplementary Fig. 1A. In addition differentiated DPCs showed positive staining for both alkaline phosphatase (Supplementary Fig. 1B) and Alizarin Red (Supplementary Fig. 1C) at both 1 and 3 wk time points. This data confirms the differentiation of DPCs toward a mineralising phenotype under our experimental conditions.

**Fig. 1:**
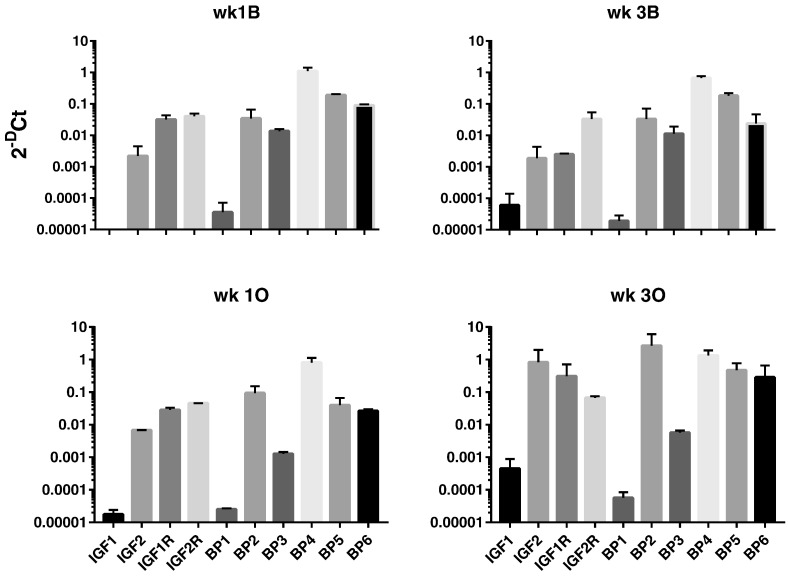
Expression of the IGF axis in DPCs (donor1): expression of IGF-1, IGF-2, IGF-1R, IGF-2R and IGFBP 1–6 after 1 wk and 3 wk incubation under basal (B) and mineralising (O) conditions relative to GAPDH are shown. Data are presented as 2^− ΔCt^ and represent the mean ± SD of triplicate technical replicates from duplicate experiments.

**Fig. 2:**
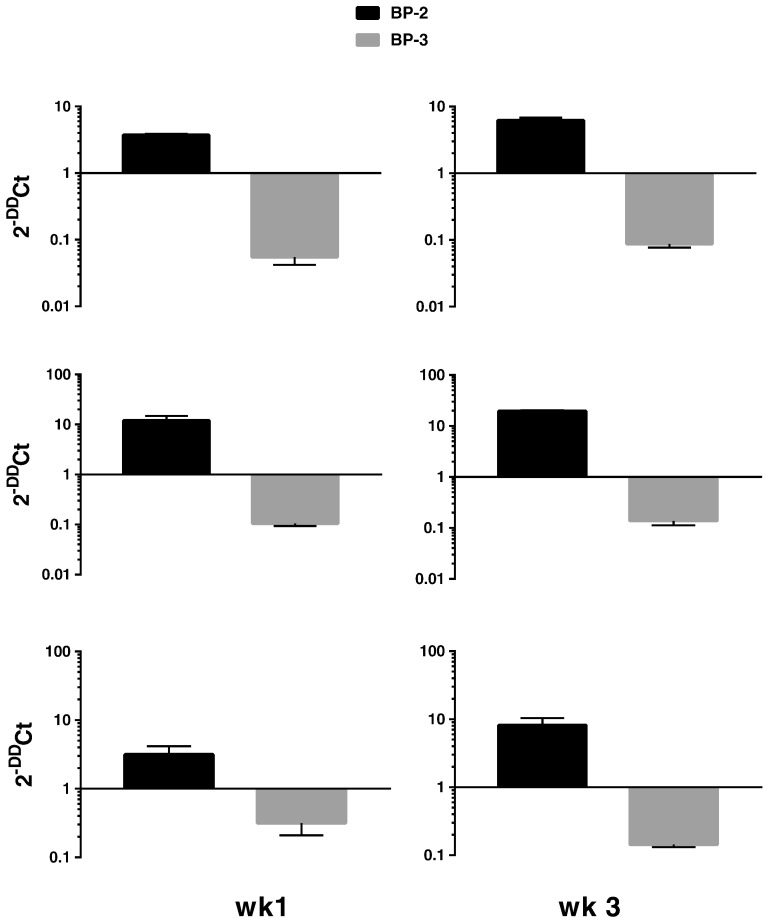
Changes in IGFBP-2 and -3 expression following the differentiation of DPCs at 1 (left panels) and 3 (right panels) wk time points. Data show fold changes in gene expression mineralising v basal culture conditions and are expressed as 2^− ΔΔCt^ representing mean ± SD of technical triplicates for cells derived from donor 1 (top panel), donor 2 (middle panel) and donor 3 (bottom panel).

**Fig. 3:**
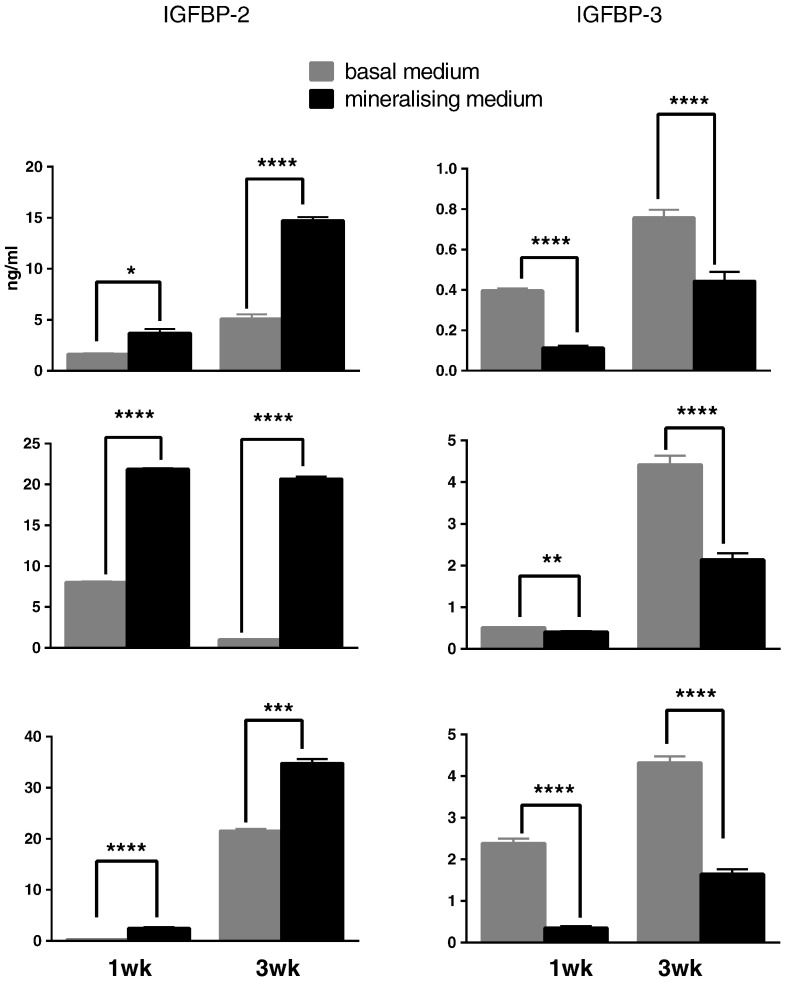
IGFBP-2 (left panels) and IGFBP-3 (right panels) concentrations in basal (grey bars) and differentiated (black bars) conditioned medium in DPCs derived from donor 1 (top panels), donor 2 (middle panels) and donor 3 (bottom panels). Data is expressed as ng/ml and represent the mean ± SD (n = 3) of duplicate or triplicate technical repeats from three independent cultures for each donor at 1 and 3 wk time points. Data was analysed by Student's unpaired *t*-test *p < 0.05 **p < 0.05 ***p < 0.001 ****p < 0.0005.

We next analysed expression of IGF axis genes (IGF-1, IGF-2, IGF-1R, IGF-2R and IGFBP1–6) in DPCs under basal and mineralising conditions. All 10 genes were expressed under both basal and mineralising conditions although in our cultures IGF-1 and IGFBP-1 were expressed at low levels Ct > 30. For IGFBPs the level of expression was IGFBP-4 > IGFBP-5 > IGFBP-6 > IGFBP-2 > IGFBP-3 > IGFBP-1 – (Fig. 1). IGFBP-4, -5 and -6 expression did not alter during differentiation of DPCs. In contrast, in each analysis of pulp cells obtained from all 3 donors we found that IGFBP-2 and IGFBP-3 expression were reciprocally regulated during differentiation at both 1 wk and 3 wk time points (Fig. 2). Induction of IGFBP-2 varied from around 4–20 fold following differentiation and similar fold reductions in IGFBP-3 expression were also seen. We also measured IGFBP-2 and -3 concentrations using ELISA (see Section 2.2) of conditioned medium (CM) from cells cultured under basal or mineralising conditions (Fig. 3). In agreement with our qRT-PCR data, IGFBP-2 protein levels in CM were elevated following differentiation of cells derived from all donors at both 1 and 3 wk time points. In most instances these differences achieved statistical significance (Fig. 3). Similarly, the decrease in IGFBP-3 mRNA expression following differentiation shown in our qRT-PCR experiments was reflected in reduced IGFBP-3 protein concentrations in conditioned cell media. For cultures derived from all donors significant decreases in IGFBP-3 concentrations were seen following differentiation of cells at both 1 and 3 wk time points Global analysis of IGFBP-2 and IGFBP-3 protein concentrations is presented in Supplementary Fig. 2, Fig. 3.

**Fig. 4:**
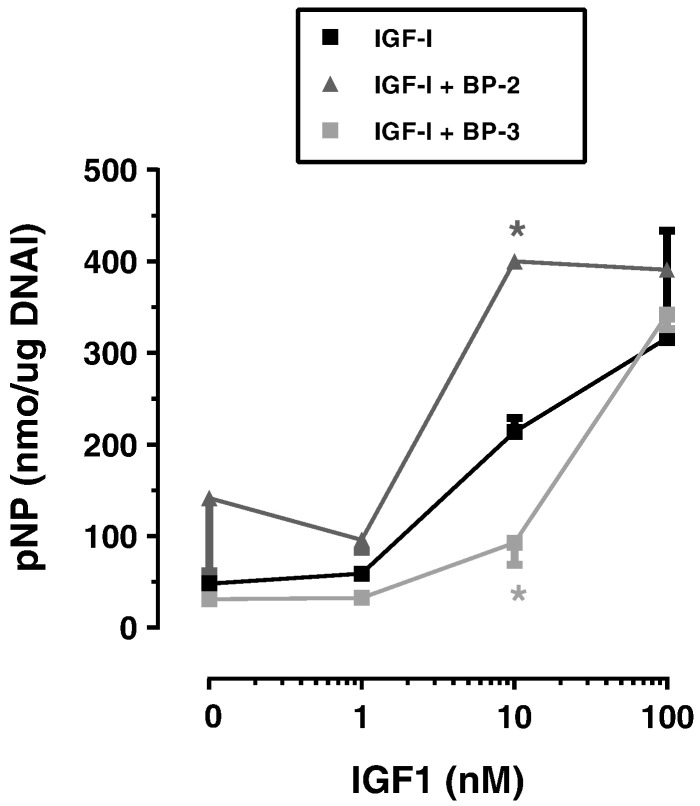
The effect of IGFBP-2 and IGFBP-3 on the mineralisation activity of exogenous IGF1. DSCs from donor 2 were incubated in mineralisation medium containing IGFs (0–100 nM) ± IGFBPs at a fixed concentration of 10 nM. Incubations were conducted for 14 days before assay of ALP activity. Data are presented as mean ± SD nmol pNP/μg DNA and represent triplicate measurement of ALP activity in two separate wells for each IGF1 concentration. In some instances symbol sizes are larger than SDs. Data was analysed by unpaired *t*-test *p < 0.01 v IGF1 alone.

To examine the functional role of IGFBP-2 and -3 we conducted the experiments described in Fig. 4 where the effects of IGFBP-2 and -3 were examined either uncomplexed or complexed with IGF1. The data indicated that IGF1 stimulated ALP activity in DPCs in a dose dependant manner and although there were no statistically significant effects of IGFBPs on their own, at equimolar concentrations of IGF: IGFBP, IGF1 activity is enhanced by IGFBP-2 and inhibited by IGFBP-3. Such effects are consistent with a functional role for both IGFBPs and are consistent with the changes in IGFBP mRNA and protein concentrations seen during the differentiation of DPCs. At higher concentrations of IGF1 maximum stimulation of ALP occurs and at this higher IGF: IGFBP molar ratio (10) the inhibitory effect of IGFBP-3 is overcome. Interestingly DPCs appeared less sensitive to stimulation by IGF2 (Supplementary Fig. 4).

## 4 Discussion

We have demonstrated reciprocal changes in IGFBP-2 and IGFBP-3 mRNA and protein expression in dental pulp stromal cells during differentiation to a mineralising phenotype and show that these changes are co-ordinated with the functions of both IGFBPs to enhance (IGFBP-2) or inhibit (IGFBP-3) the mineralising activity of IGF1 in these cells. Although IGF1 has been reported previously to promote the differentiation of human dental pulp stem cells via mTor (Feng et al., 2014) and MAPK/Stat-3 (Vandomme et al., 2014) signalling pathways there is only a limited literature describing IGFBP-2 and/or IGFBP-3 expression and activity during osteogenic differentiation. A direct effect of IGFBP-2 on the osteoblastic differentiation of rat calvarial cells via a receptor tyrosine phosphatase β (RPTPβ) based mechanism was recently reported (Xi et al., 2014). Whether such a mechanism is associated with the potentiation of IGF1 activity by IGBP-2 reported in the current study is unknown. A potentiating effect of IGFBP-2 on IGF-2 stimulation of alkaline phophatase activity in differentiating rat tibial osteoblast cultures (Palermo et al., 2004) was reported although we found that DPCs were less sensitive to IGF2 stimulation compared to IGF1 in terms of stimulation of ALP activity (Supplementary Fig. 4). This suggests that the pro-mineralising action of IGFs is principally signalled through the IGF1R although confirmation of this should be sought through the use of specific IGF1R inhibitors. There are two reports which demonstrate upregulation of IGFBP-5 expression during mineralisation of dental pulp cells. Microarray analysis indicated an 8-fold increase in IGFBP-5 expression in DPCs derived from non-carious wisdom teeth following 10 days treatment with mineralisation medium although this group did not confirm IGFBP-5 protein expression (Mori et al., 2011, Mori et al., 2010). Although we found that IGFBP-5 was expressed under both basal and mineralising conditions in DPCs we did not find any changes in gene expression under our experimental conditions in any of our donors. Although it is difficult to reconcile these results factors such as differences in precise tissue culture conditions or donor profiles may be involved.

The IGF axis may also play an indirect role(s) in the differentiation of dental tissues. For example IGF-1 increased extra-cellular matrix secretion by dental-pulp derived fibroblasts (Nakashima, 1992) via the induction of bone morphogenetic protein (BMP)-2 expression (Li et al., 1998). Although our qRT-PCR data indicated a low abundance of IGF-1 mRNA expression in DPCs, in vivo dental pulp tissue is well vascularised and would therefore have access to systemic IGF1 and our data would suggest that growth factor activity could be modulated by locally expressed IGFBP-2 and -3. Conversley IGF-2 and IGF-1R were expressed at moderate to high levels in our experiments confirming previous reports (Caviedes-Bucheli et al., 2004, Shi et al., 2001). Both IGF-1 and IGF-2 (acting via the IGF-1R) can induce ALP activity in canine dental pulp cells (Onishi et al., 1999) and IGF-2 secretion was reported during matrix mineralisation of human dental pulp derived fibroblasts (Reichenmiller et al., 2004). IGF axis components are present in other dental structures with IGF-1 and -2 along with all six IGFBPs present in the ECM of the periodontal ligament and IGF-1R present on the surface of periodontal ligament derived fibroblasts (Gotz et al., 2006). A very recent study using stem cell populations isolated from apical papillae (SCAP) reported the stimulation of cell proliferation, ALP expression and mineralisation activity by IGF-1. Simultaneously, expression of odontogenic markers (dentin sialoprotein and dentin sialophosphoprotein) was downregulated arguing for a bias in IGF-1 action toward bone formation and away from odontogenic differentiation in this tissue niche (Wang et al., n.d.). However it should be noted that microscopic confirmation of appropriate bone tissue structure was not rigorously established in these studies and this area requires further investigation. In a detailed study of IGF axis expression in differentiating ameloblasts, position specific expression of IGF-1, IGF-2, IGF-1R and IGF-2R toward the outer enamel layer and away from pulp facing ameloblasts was reported arguing for the importance of the IGF axis in development of this tissue (Caviedes-Bucheli et al., 2009, Yamamoto et al., 2006) and an elegant study demonstrating the developmental stage-dependent expression of IGF-1 in the continually erupting rat incisor model (Joseph et al., 1996) also argues strongly for a role of the IGF axis in the development of dental tissues.

In our current study IGFBP-2 levels were approximately 10-fold higher than those of IGFBP-3 suggesting that the large induction of IGFBP-2 may play a more import role in the differentiation of DPCs than the decrease seen in IGFBP-3 concentrations. Although an extensive literature describes both IGF-dependent and IGF independent effects of IGFBPs – reviewed in Annunziata et al. (2011) - in most instances quantitative aspects related to IGF and IGFBP concentrations in the local tissue environment have largely been ignored. Our data suggest that the ratio of IGF:IGFBP present in an experimental model can be important in determining the overall biological response (Fig. 4). These factors are also important with respect to the functional redundancy displayed by IGFBPs in some tissue culture and whole animal studies (Murphy, 1998, Pintar et al., 1995) and the accurate quantification of local IGF axis proteins in vivo or in vitro is essential for an understanding of biological outcomes. Notwithstanding these arguments evidence suggests an important role for the IGF axis in the differentiation and development of various dental structures. However there are several gaps in our knowledge of the role of various members of this molecular axis (particularly IGFBPs) in this process and it is hoped that future studies will shed some light in this area and also assist in design of strategies aimed at hard tissue engineering using multipotent stem cells derived from dental pulp and other stem cell niches within the oral cavity.

## Appendix A Supplementary data

Supplementary Fig. 1 (A) qRT-PCR analysis of ALP, RunX2 and OCN expression in DPCs cultured under matrix mineralisation conditions. Data are presented as 2^− ΔΔCt^ and represent fold changes in gene expression at 1 and 3 wk time points. (B) ALP staining of DPCs cultured in monolayers under basal and mineralising conditions for 1 and 3 weeks as indicated Scale bar = 5 cm. (C) Mineralised nodules are stained positively (red) with Alizarin red cultured in monolayers under mineralising conditions for 1 and 3 wk as labelled. Scale bar = 100 μm. Supplementary Fig. 2 Global analysis of IGFBP-2 concentrations in DPCs in basal (B) and osteogenic (O) conditioned medium. Data is shown for DPCs at both 1 (upper panel) and 3 (lower panel) wk time points and represents mean ± SD (n = 3) of triplicate technical replicates from duplicate cultures for each of three donors. *p < 0.05. Supplementary Fig. 3 Global analysis of IGFBP-3 concentrations in DPCs in basal (B) and osteogenic (O) conditioned medium. Data is shown for DPCs at both 1 (upper panel) and 3 (lower panel) wk time points and represents mean ± SD (n = 3) of triplicate technical replicates from duplicate cultures for each of three healthy donors. *p < 0.05. Supplementary Fig. 4 The effect of IGF2 on ALP activity in DPCs grown under mineralising conditions. IGF2 was added at 0-100nM and assays were terminated at 2 wk as described in Methods. *p < 0.05 v 0 nM IGF2. Table SI Donor profiles. Table SII Assay identifiers for TaqMan qRT-PCR: further details are available at www.appliedbiosystems.com. Image 1
