# Supplementary material for: IGFBP-2 and -3 co-ordinately regulate IGF1 induced matrix mineralisation of differentiating human dental pulp cells
Source: Stem Cell Res. 2016 Nov;17(3):517–22. doi: 10.1016/j.scr.2016.09.026 (PMC5153425; doi:10.1016/j.scr.2016.09.026)

Supplementary Table I

Donor	Tooth	Age	Gender	Ethnic Origin
1	Healthy third molar	20	Female	Asian
2	Healthy third molar	35	Female	Cauc.

3	Healthy third molar	24	Female	Cauc.

Supplementary Table II

Gene Name	***TaqMan®Gene expression assay identifier***
GAPDH	Hs99999905_m1
ALPL	Hs01029144_m1
OCN	Hs00609452_g1
Runx2	Hs00231692_m1
IGF1	Hs01547656_m1
IGF2	Hs04188276_m1
IGF1R	Hs00609566_m1
IGF2 R	Hs00974474_m1
IGFBP 1	Hs00236877_m1
IGFBP 2	Hs01040719_m1
IGFBP 3	Hs00426289_m1
IGFBP4	Hs01057900_m1
IGFBP 5	Hs00181213_m1
IGFBP 6	Hs00181853_m1

Figure 1S

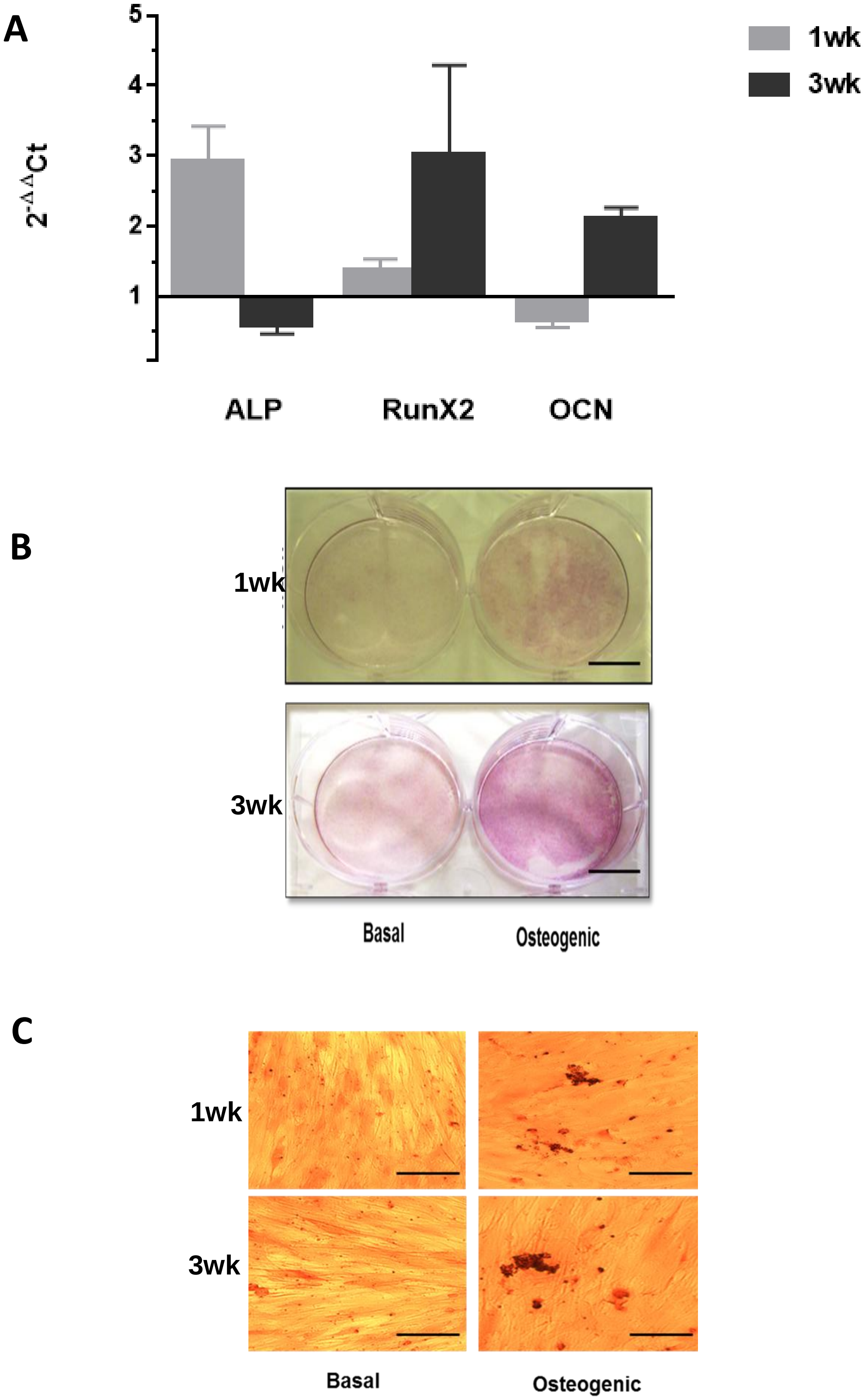

A
$2^{-\Delta\Delta}$Ct
5
4
3
2
1
1wk
3wk
ALP
RunX2
OCN
B
1wk
3wk
Basal
Osteogenic
C
1wk
3wk
Basal
Osteogenic

Figure 2S

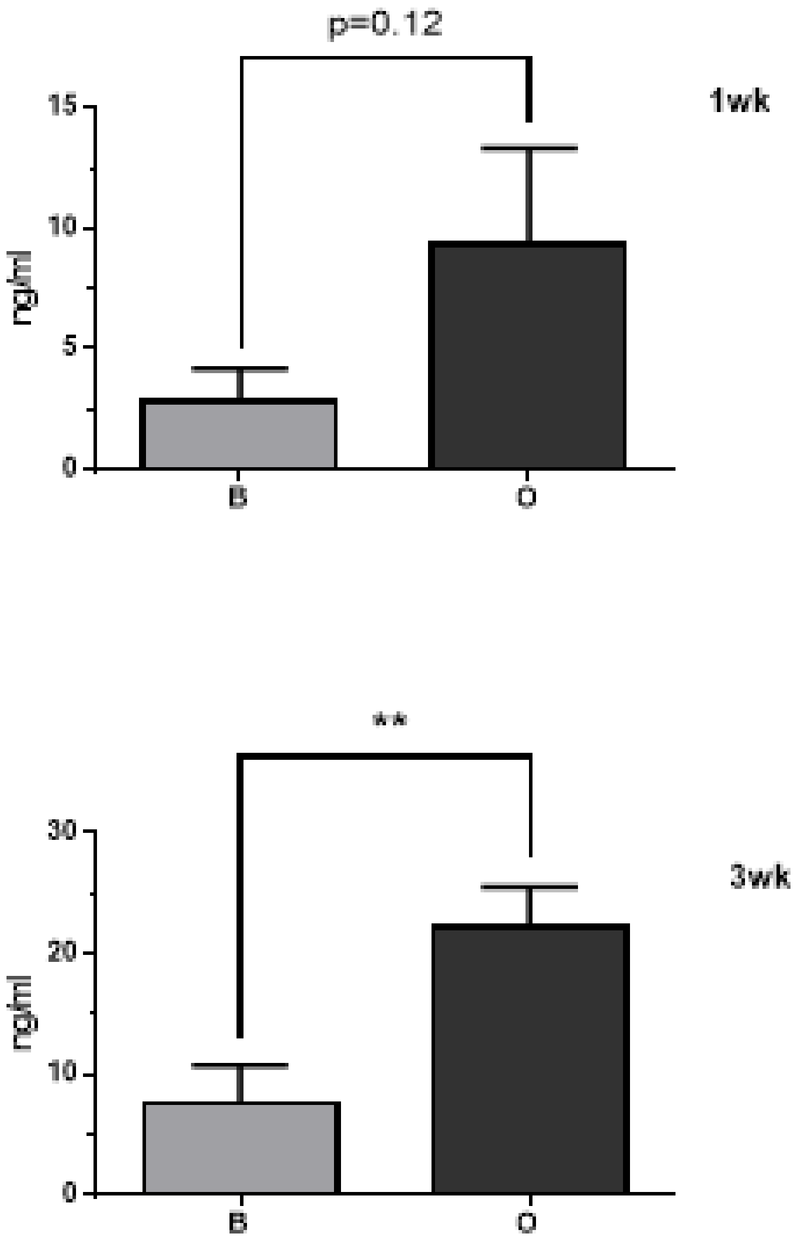

Figure 3S

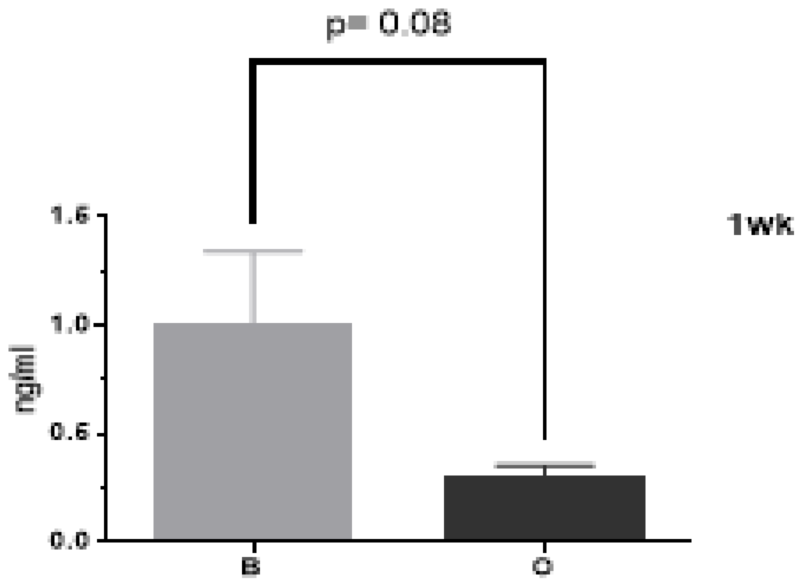

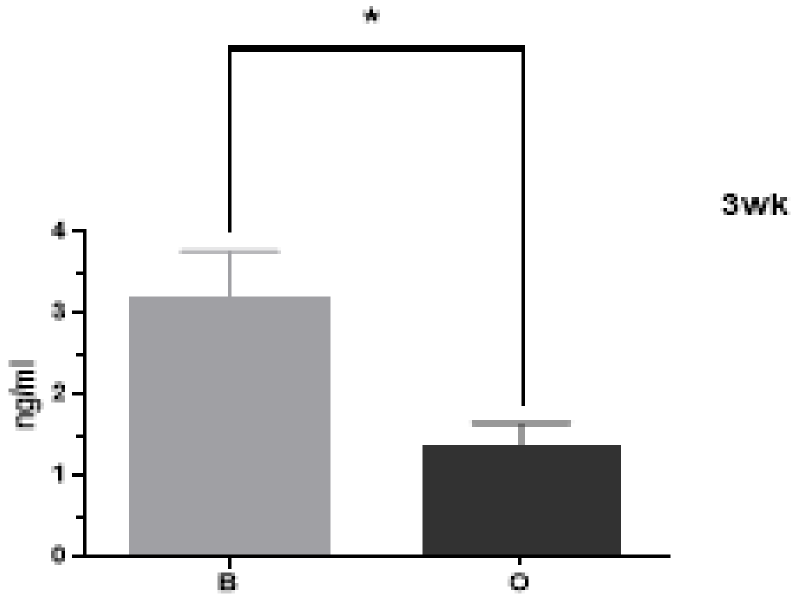

Figure 4S

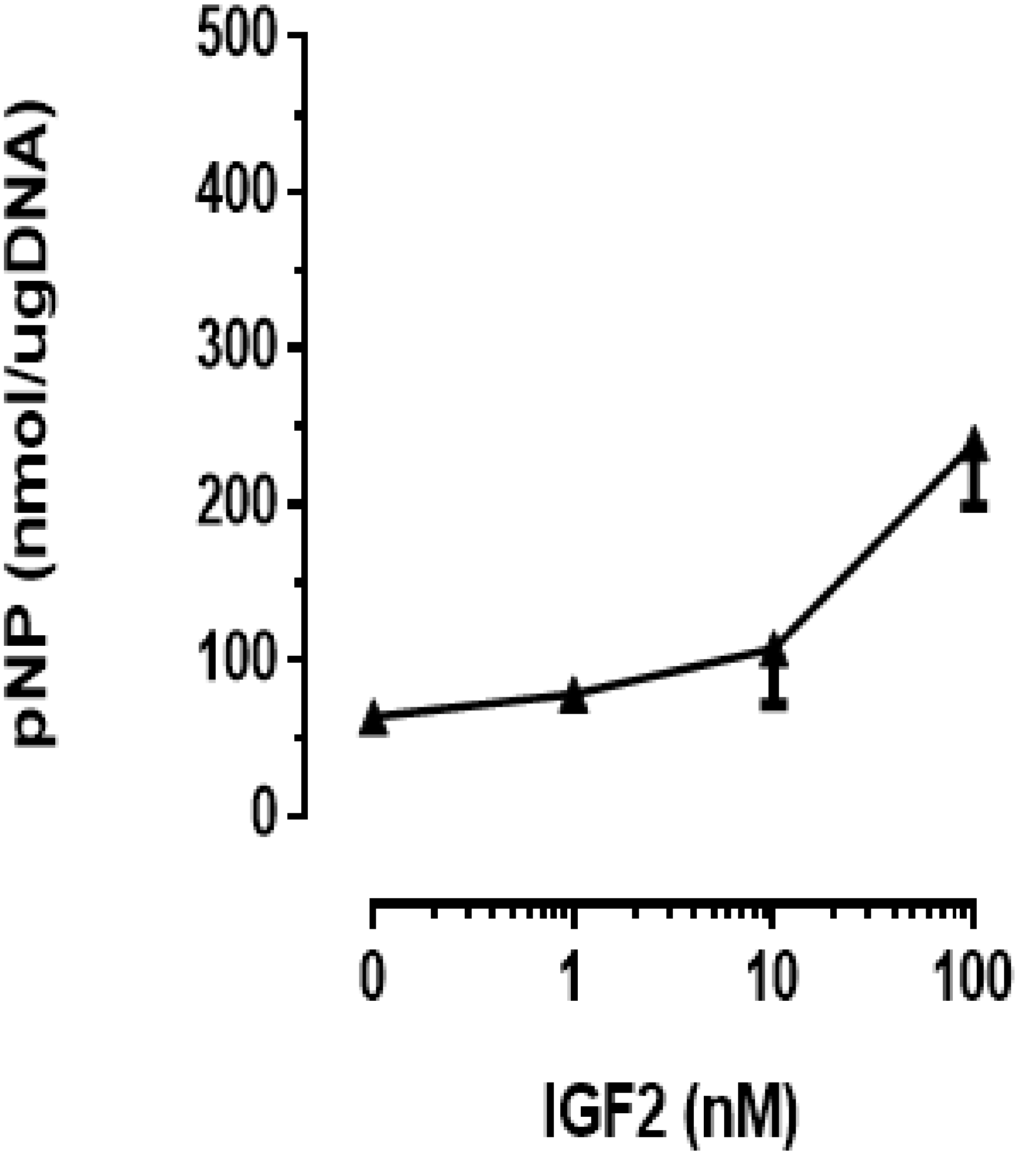

Supplement: Supplementary file 1 — Supplementary Fig. 1 (A) qRT-PCR analysis of ALP, RunX2 and OCN expression in DPCs cultured under matrix mineralisation conditions. Data are presented as 2− ΔΔCt and represent fold changes in gene expression at 1 and 3 wk time points. (B) ALP staining of DPCs cultured in monolayers under basal and mineralising conditions for 1 and 3 weeks as indicated Scale bar = 5 cm. (C) Mineralised nodules are stained positively (red) with Alizarin red cultured in monolayers under mineralising conditions for 1 and 3 wk as labelled. Scale bar = 100 μm. Supplementary Fig. 2 Global analysis of IGFBP-2 concentrations in DPCs in basal (B) and osteogenic (O) conditioned medium. Data is shown for DPCs at both 1 (upper panel) and 3 (lower panel) wk time points and represents mean ± SD (n = 3) of triplicate technical replicates from duplicate cultures for each of three donors. *p < 0.05. Supplementary Fig. 3 Global analysis of IGFBP-3 concentrations in DPCs in basal (B) and osteogenic (O) conditioned medium. Data is shown for DPCs at both 1 (upper panel) and 3 (lower panel) wk time points and represents mean ± SD (n = 3) of triplicate technical replicates from duplicate cultures for each of three healthy donors. *p < 0.05. Supplementary Fig. 4 The effect of IGF2 on ALP activity in DPCs grown under mineralising conditions. IGF2 was added at 0-100nM and assays were terminated at 2 wk as described in Methods. *p < 0.05 v 0 nM IGF2. Table SI Donor profiles. Table SII Assay identifiers for TaqMan qRT-PCR: further details are available at www.appliedbiosystems.com. [file mmc1.pdf]
